# Supplementary figures and images for: The microbiome compositional and functional differences between rectal mucosa and feces
Source: Microbiol Spectr. 2024 Jun 25;12(8):e03549-23. doi: 10.1128/spectrum.03549-23 (PMC11302734; doi:10.1128/spectrum.03549-23)

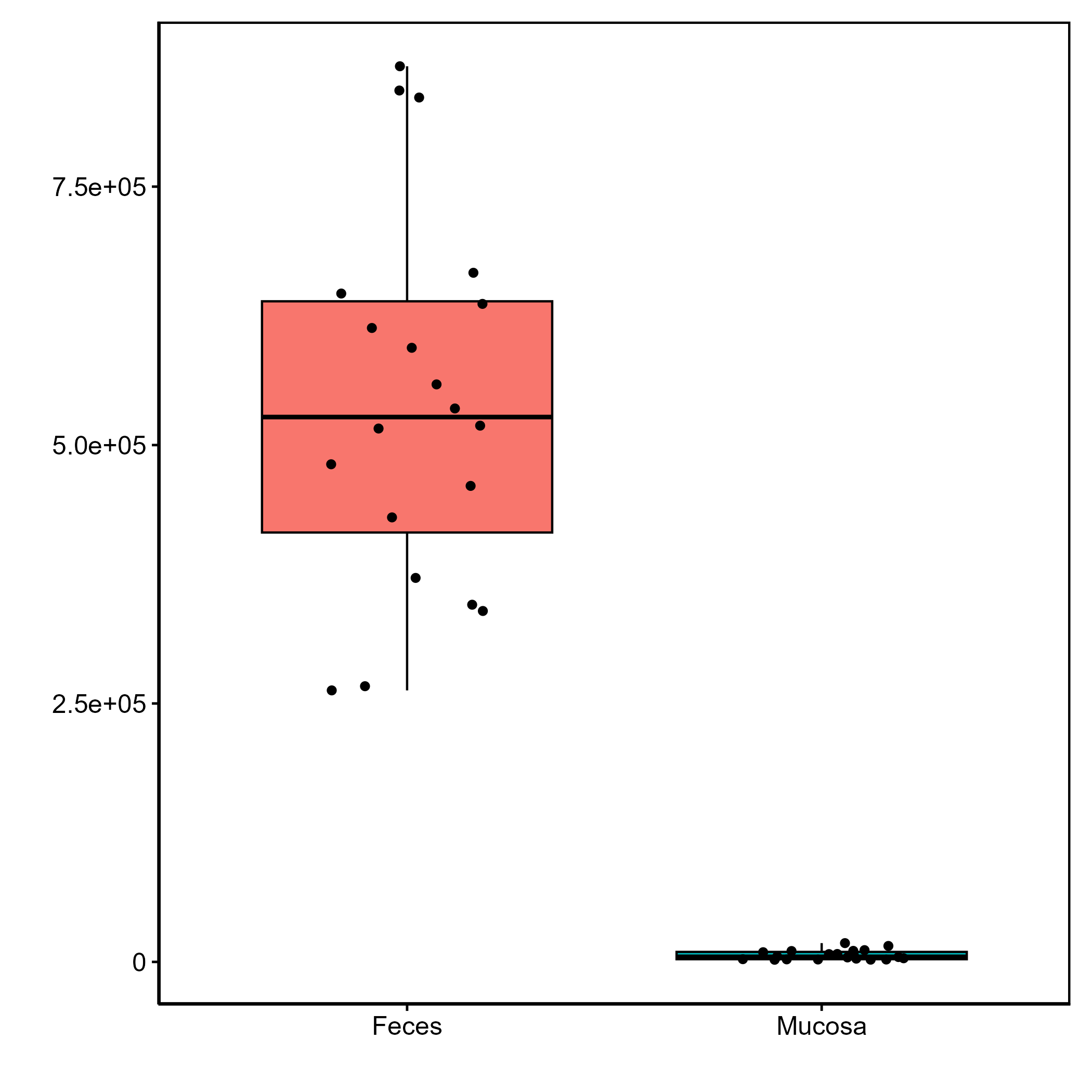

Supplement: Fig. S1 — Detected microbial genes. [file spectrum.03549-23-s0001.tif]

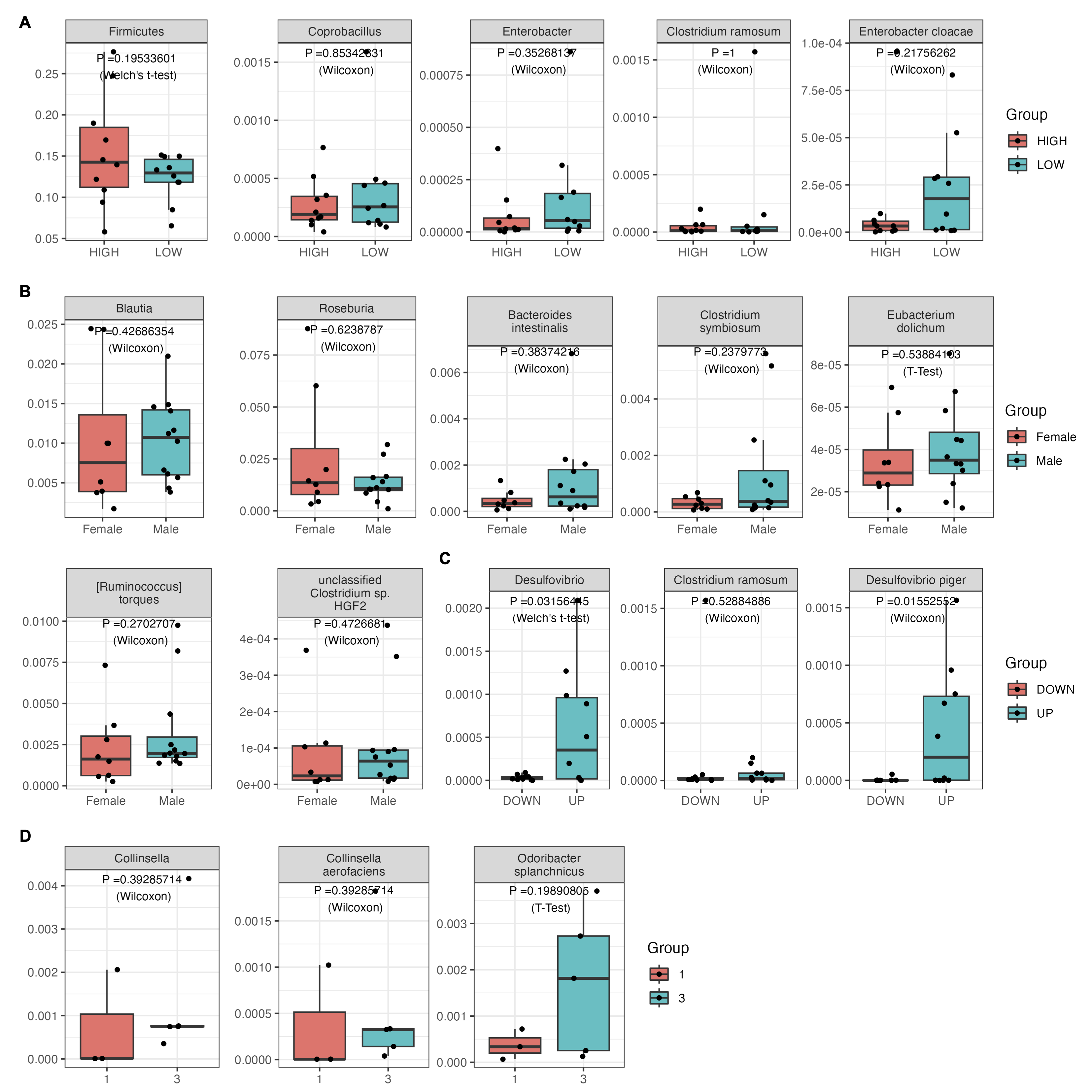

Supplement: Fig. S2 — Relative abundance of phenotype-associated differential biomarkers. [file spectrum.03549-23-s0002.tif]

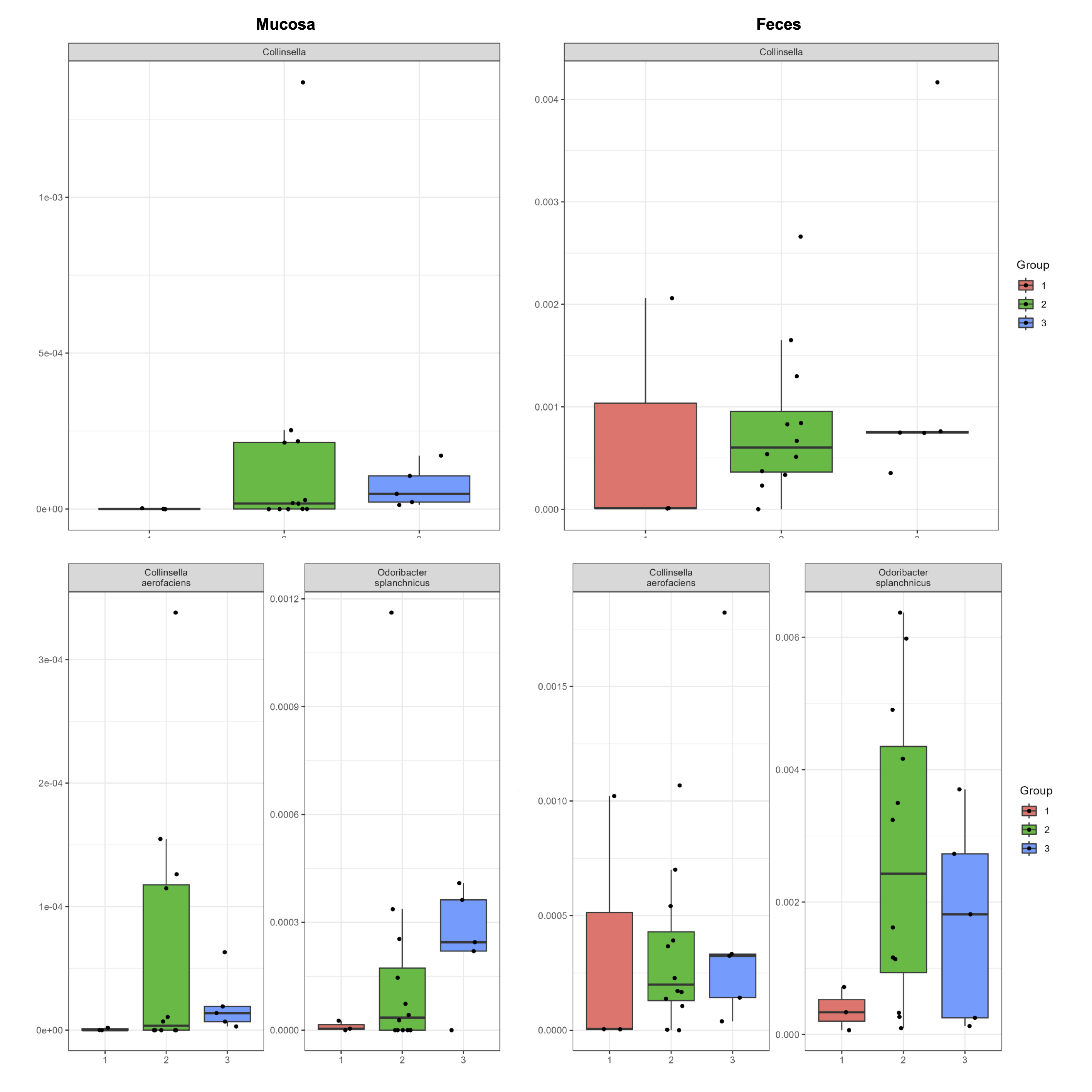

Supplement: Fig. S3 — Relative abundance distribution of the three taxonomic groups. [file spectrum.03549-23-s0003.tif]
