# Supplementary material for: The microbiome compositional and functional differences between rectal mucosa and feces
Source: Microbiol Spectr. 2024 Jun 25;12(8):e03549-23. doi: 10.1128/spectrum.03549-23 (PMC11302734; doi:10.1128/spectrum.03549-23)
Supplement: Supplemental material — Legends for Fig. S1 to S3. [file spectrum.03549-23-s0004.docx]

The microbiome compositional and functional differences between rectal mucosa and feces

Supplementary Figures


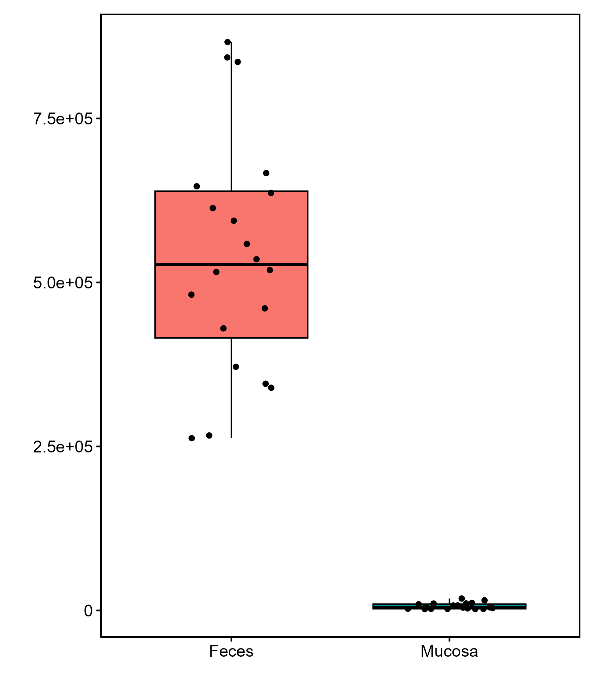


**Supplementary Fig. 1** Total number of detected microbial genes (gene richness) for rectal mucosa and feces.


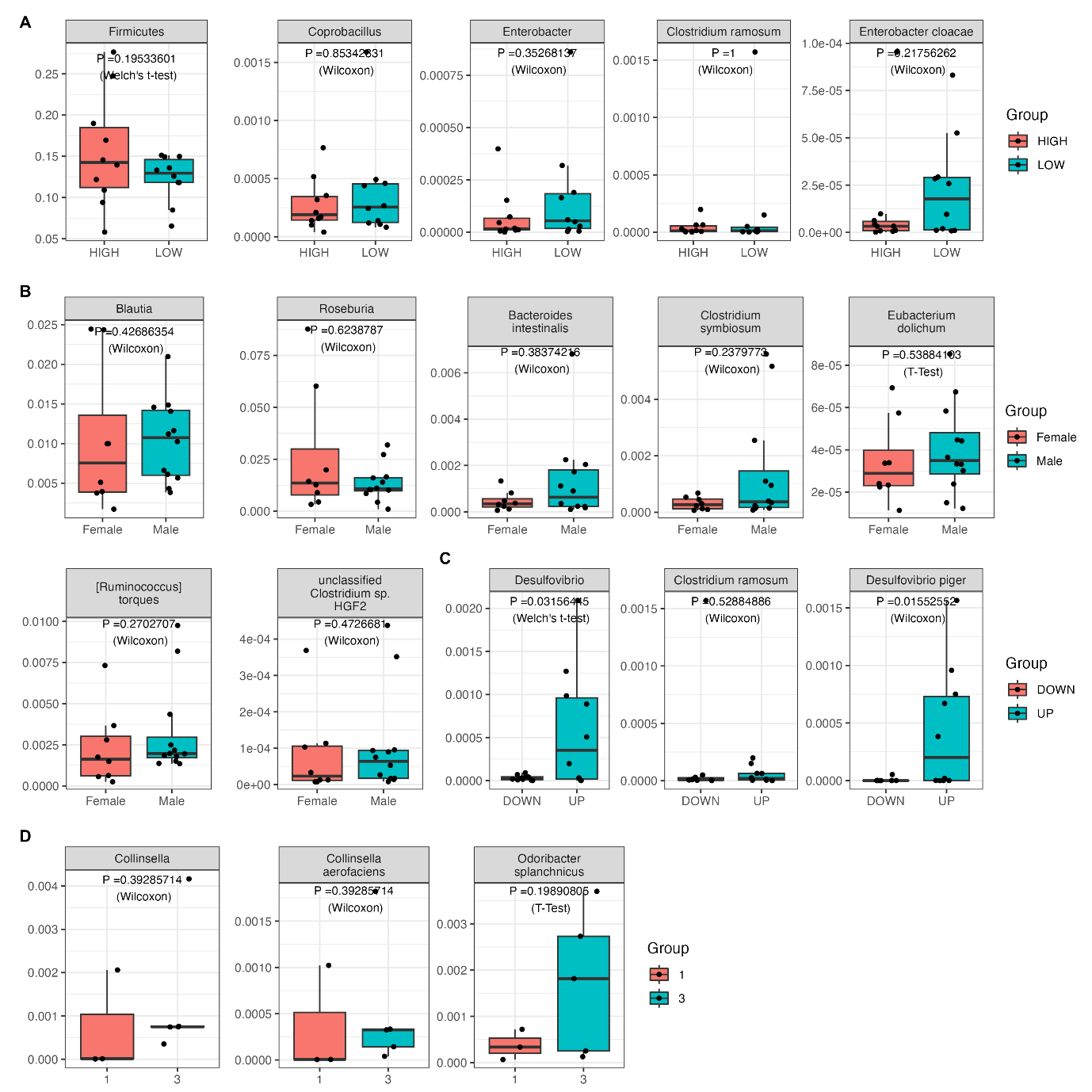


**Supplementary Fig. 2** Relative abundance of phenotype-associated differential biomarkers in the rectal mucosa in corresponding fecal samples. (A) Age. (B) Gender. (C) BMI. (D) Polyp risk.

**
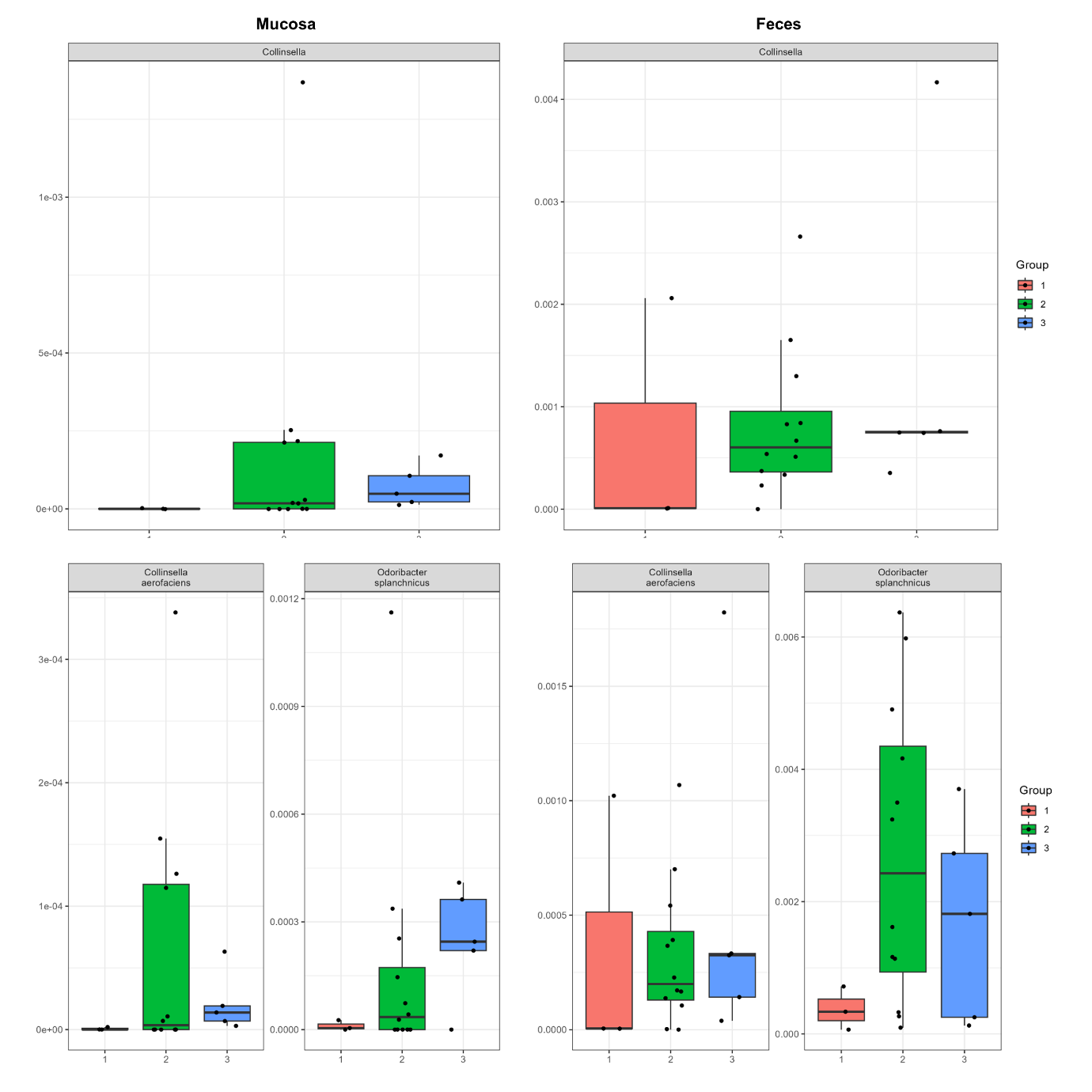
**

**Supplementary Fig. 3** The relative abundance distribution of the three taxonomic groups across three polyp risk levels, as well as in rectal mucosa and feces.
